# Genetic and Proteomic Characterization of *rpoB* Mutations and Their Effect on Nematicidal Activity in *Photorhabdus luminescens* LN2

**DOI:** 10.1371/journal.pone.0043114

**Published:** 2012-08-17

**Authors:** Xuehong Qiu, Xun Yan, Mingxing Liu, Richou Han

**Affiliations:** Guangdong Entomological Institute, Guangzhou, Guangdong, China; University of South Florida College of Medicine, United States of America

## Abstract

Rifampin resistant (Rif^R^) mutants of the insect pathogenic bacterium *Photorhabdus luminescens* LN2 from entomopathogenic nematode *Heterorhabditis indica* LN2 were genetically and proteomically characterized. The Rif^R^ mutants showed typical phase one characters of *Photorhabdus* bacteria, and insecticidal activity against *Galleria mellonella* larvae, but surprisingly influenced their nematicidal activity against axenic infective juveniles (IJs) of *H. bacteriophora* H06, an incompatible nematode host. 13 out of 34 Rif^R^ mutants lost their nematicidal activity against H06 IJs but supported the reproduction of H06 nematodes. 7 nematicidal-producing and 7 non-nematicidal-producing Rif^R^ mutants were respectively selected for *rpoB* sequence analysis. *rpoB* mutations were found in all 14 Rif^R^ mutants. The *rpoB* (P564L) mutation was found in all 7 mutants which produced nematicidal activity against H06 nematodes, but not in the mutants which supported H06 nematode production. Allelic exchange assays confirmed that the Rif-resistance and the impact on nematicidal activity of LN2 bacteria were conferred by *rpoB* mutation(s). The non-nematicidal-producing Rif^R^ mutant was unable to colonize in the intestines of H06 IJs, but able to colonize in the intestines of its indigenous LN2 IJs. Proteomic analysis revealed different protein expression between wild-type strain and Rif^R^ mutants, or between nematicidal-producing and non nematicidal-producing mutants. At least 7 putative proteins including DsbA, HlpA, RhlE, RplC, NamB (a protein from T3SS), and 2 hypothetical proteins (similar to unknown protein YgdH and YggE of *Escherichia coli* respectively) were probably involved in the nematicidal activity of LN2 bacteria against H06 nematodes. This hypothesis was further confirmed by creating insertion-deletion mutants of three selected corresponding genes (the downregulated *rhlE* and *namB*, and upregualted *dsbA*). These results indicate that the *rpoB* mutations greatly influence the symbiotic association between the symbionts and their entomopathogenic nematode hosts.

## Introduction

Rifampin (Rif), first introduced in 1972 as an antitubercular drug, was initially extremely effective against *Mycobacterium tuberculosis*, and other bacteria [1]–[2]. With its widespread and extended use, the number of bacterial isolates resistant to Rif has increased. Most Rif-resistance mutations in *M. tuberculosis* as well as in *Escherichia coli* and *Staphylococcus aureus* were conferred by a set of restrictive mutations in the *rpoB* gene, which encoded the β-subunit of RNA polymerase (RNAP) in bacteria [3]–[4]. DNA-dependent RNAP, which contains an essential catalystic core enzyme (α_2_ββ’ω) and one of the sigma (δ) factors, is the central enzyme for expression of genomic information in all organisms. Rif inhibits transcription initiation by blocking the *rpoB* of bacterial RNAP [5]–[6]. *E. coli rpoB* mutations that suppress the auxotrophy due to lack of stringent response were demonstrated to affect the transcription of stringently controlled genes by destabilizing the RNAP-stable RNA promoter complex [7]. The Rif resistant (Rif^R^) *M. tuberculosis* mutations of the *rpoB* gene were found in nearly 95% of clinical isolates [4]. Most of the mutations were located from nucleotides 1276 to 1356 (codon 432–458 in *M. tuberculosis rpoB* gene and codon 507–533 in *E. coli rpoB* gene). An 81 bp core region was called the Rif resistance determining region (RRDR) of *rpoB* [8]–[10]. However, a significant number of Rif^R^ mycobacteria with no mutations in the *rpoB* gene have been isolated from different clinical samples [11]–[14]. A 191A/C mutation in the Rv2629 gene was reported to be significantly associated with Rif-resistance in *M*. *tuberculosis* [15]. Recently, it was reported that the K1 uptake regulator TrkA played an important role in intrinsic and acquired antibiotic resistance in mycobacteria [16]. Besides Rif-resistance, the *rpoB* mutation (A621E) conferred dual heteroresistance to daptomycin and vancomycin in *Staphylococcus aureus* [17], but most *rpoB* mutations were involved in reduced vancomycin susceptibility [18]. It suggested that different *rpoB* mutations may have different effect on bacteria.

*Photorhabdus* and *Xenorhabdus* bacteria belonging to the Enterobacteriaceae are symbiotically associated respectively with entomopathogenic *Heterorhabditis* and *Steinernema* nematodes, which are used as a commercial bioinsecticide for many economically important insect pests [19]. The association between the nematodes and their symbiotic bacteria plays an important role in the pathogenicity and production of these nematode-bacterium complexes. The infective juveniles (IJs) of these nematodes are a developmentally arrested non-feeding form, ensheathed in the second stage cuticle and harbor *Photorhabdus* or *Xenorhabdus* cells as symbionts in their intestines. The IJ nematodes properly maintain and carry the bacteria needed for killing insects and providing a suitable environment for the reproduction of new vectors [20]–[22]. Different *Photorhabdus* or *Xenorhabdus* bacterial isolates differ in their ability to support *in vitro* monoxenic cultures of non-host nematodes [23]–[30] and to retain the bacterial cells in the IJ intestines [22]–[23], [26], [28], [31].

Strains of *Photorhabdus* and *Xenorhabdus* not only show insecticidal activities towards different insects [32]–[36] but also exhibit nematicidal activities against some plant nematodes, such as *Meloidogyne incognita* [37], the free-living soil nematode *Caenorhabditis elegans* [38], and for *Steinernema* nematodes [39].

The trans-specific nematicidal activity of *P. luminescens* subsp. *akhurstii* LN2, a normal symbiont of *H. indica* LN2, against *H*. *bacteriohora* H06 nematodes was previously observed [40]. These bacteria secrete unidentified toxic factors lethal for H06 nematodes although the bacteria produce signals which trigger the recovery of H06 IJ nematodes [30]. A novel *P. luminescens* LN2 gene involved in the nematicidal activity against *H. bacteriophora* H06 IJs was identified [41].

*Xenorhabdus* and *Photorhabdus* bacterial isolates resistant to Rif were used in several references [42]–[46]. When different Rif^R^ mutants of *P. luminescens* LN2 were monoxenically combined respectively with the axenic IJs of a Chinese isolate *H. bacteriophora* H06, the involvement of *rpoB* mutation in the nematicidal activity (incompatible symbiosis) was discovered.

To achieve an overall view of phenotypic, genetic and metabolic modifications associated with different Rif^R^ mutants, the experiments were conducted to determine: (1) the effects of different Rif^R^ mutants of *P. luminescens* LN2 on the growth of their corresponding incompatible nematode hosts, *H. bacteriophora* H06; (2) the phenotypic and biochemical characters of the Rif^R^ mutants; (3) the *rpoB* mutations in the Rif^R^ mutants; (4) the effects of *rpoB* mutations in the Rif^R^ mutants on the nematode growth; (5) the mutualistic colonization of H06 IJs by the Rif^R^ mutants; (6) the proteomic analysis of the mutants and wild-type bacterial strain; (7) the effects of differentially expressed proteins detected from proteomic analysis on nematicidal activity.

## Materials and Methods

### Nematode Species, Bacterial Strains, Plasmids and Culture Conditions

Bacterial strains and plasmids used in this study are listed in Table 1. *P. luminescens* subsp. *akhurstii* LN2 isolated from its host nematode *H. indica* LN2 was used for the isolation of spontaneous Rif^R^ mutants. *P. luminescens* H06 or HNA were used for the mass production of *H. bacteriophora* H06. The bacterial strains were cultured in LB1 broth (1% tryptone, 0.5% yeast Extract, 0.5% NaCl) or on LB1 agar at 25°C. The primary form (phase one) of these bacteria was obtained by selecting green or blue-green colonies on NBTA or red colonies on MacConkey agar, and repeated subculturing [47]. Stock cultures were maintained in 15% glycerol (v/v) in LB1 at −80°C. *E. coli* strains were grown in LB2 broth (1% tryptone, 0.5% yeast Extract, 1% NaCl) or on LB2 agar at 37°C.

**Table 1:** Bacterial strains and plasmids used in this study.

Strain/plasmid	Description, relevant characteristics	Reference or source
*Photorhabdus luminescens* strains		
LN2/LN2-W	Wild-type isolate from host nematode *H. indica* LN2	Ralf-Udo Ehlers
LN2-R1∼LN2-R34	Spontaneous Rif^R^ mutant of wild-type strain LN2; Rif^R^	This study
LN2-A	Spontaneous Amp^R^ mutant of wild-type strain LN2; Amp^R^	This study
LN2-M1	Tn*5* insertion mutant of LN2; Amp^R^, Km^R^	[41]
H06	Wild-type isolate from host nematode *H. bacteriophora* H06	Laboratory stock
HNA	Wild-type isolate from host nematode *H. megidis* HNA	Dr Wim Wouts
LN2-WΔ*rpoB*-LR31	LN2-W containing the mutant *rpoB* allele from LN2-R31	This study
LN2-R31Δ*rpoB*-LW	LN2-R31containing the wild-type *rpoB* allele from LN2-W	This study
LN2Δ*rhlE*	LN2-A *rhlE*::Cm	This study
LN2Δ*namB*	LN2-A *namB*::Cm	This study
LN2Δ*dsbA*	LN2-A *dsbA*::Cm	This study
*Escherichia coli* strains		
DH5a	Host of plasmids	TaKaRa
S17-1 (*λpir*)	*E. coli* lysogenized with *λpir*, replication of *ori* R6K	[51]
TOP10	Cloning strain	Invitrogen
Plasmids		
pMini-Tn*5*	*ori*T, *ori*R6K, delivery plasmid for mini-Tn*5*; Km^R^	[52]
pCR4-TOPO	Cloning vector; Amp^R^, Km^R^	Invitrogen
pMD-18T	Cloning vector; Amp^R^	TaKaRa
pMD-19T	Cloning vector; Amp^R^	TaKaRa
pUC19-egfp	pUC19 carrying *egfp*; Amp^R^	Laboratory stock
pMD-egfp	pMD-18T carrying ribosome binding site and *egfp* gene; Amp^R^	This study
pMD-lac-egfp	pMD-19T carrying ribosome binding site, *lacZ* promoter and *egfp* gene; Amp^R^	This study
pMini-lac-egfp	pMini-Tn*5* carrying a *Not*I fragment containing ribosome binding site, *lacZ* promoter and *egfp* gene; Km^R^	This study
pPHU281	*lacZ’ mob*(RP4), Tc^R^ derivative of pUC18 with *oriT*	[50]
pPHU281-rpoB-LW	pPHU281 carrying a *Bam*HI-*Pst*I fragment containing *rpoB* gene from wild-type strain of *P. luminescens* LN2	This study
pPHU281-rpoB-LR31	pPHU281 carrying a *Bam*HI-*Pst*I fragment containing *rpoB* gene from Rif^R^ mutant of *P. luminescens* LN2-R31	This study
pKNG101	R6K Ori, *sacB*, Sm^R^	[56]
pKNG101-*rhlE*::Cm	pKNG101 carrying a *rhlE*::Cm fragment, Sm^R^, Cm^R^	This study
pKNG101- *namB*::Cm	pKNG101 carrying a *namB*::Cm fragment, Sm^R^, Cm^R^	This study
pKNG101- *dsbA*::Cm	pKNG101 carrying a *dsbA*::Cm fragment, Sm^R^, Cm^R^	This study

When required, antibiotics were added to the medium with the following concentrations: ampicillin (Amp), 100 µg/mL; kanamycin (Km), 50 µg/mL; rifampin (Rif), 50 µg/mL; and tetracycline (Tc), 25 µg/mL; chloramphenicol (Cm), 25 µg/mL. All the antibiotics used in this study were purchased from Sigma Chemical Company and all medium components from Oxoid Company, England.

### Production of Axenic *Heterorhabditis* IJs

Axenic *H. bacteriophora* H06 IJs for the monoxenic nematode-bacterium recombinations were obtained according to the method as previously described [30]. Briefly, IJs of H06 were grown monoxenically on nonspecific *P. luminescens* HNA on a sponge medium consisting of 1% yeast extract, 5% egg yolk, 15% soya flour, 5% corn oil, 8% polyether polyurethane sponge and 50% distilled water [30]. The IJs were collected by centrifugation and migration through a 30 µm nylon cloth sieve under sterile conditions, surface-sterilized in 0.5% streptomycin-sulfate (Merck, Germany) for 6 h and then rinsed three times in sterile distilled water. The axenicity of these surface-sterilized IJs was checked as previously described [30]. Because these IJs can be reared with the provided bacterial isolates, and are not able to contain the bacteria in their intestines, they are free of bacteria after surface sterilization.

### Nematicidal Bioassay of the Rif^R^ Mutants

The Rif^R^ mutants from LB1 agar with Rif were screened for their nematicidal activities against *H. bacteriophora* H06 IJs according to the method as previously described [30]. Approximately 100 axenic H06 IJs were introduced to the 2-day old lawn of wild-type strain or Rif^R^ mutants of *P. luminescens* LN2 grown on LB1 agar in 96-well tissue culture plate (Corning, New York, USA). Mortality and growth of the IJs were observed daily and recorded until 15 days. A lawn of wild-type *P. luminescens* H06 was used as a control. A mutant was considered positive for nematode growth if the tested nematodes were able to survive at least 7 days and produce the next generation of juveniles from the hermaphrodites. If the mutants were unable to support the survival of nematodes, the introduced IJs died after 7 days. The effect of the Rif^R^ mutants on H06 IJs were verified by repeating the nematode survival and growth experiments three times, each with 12 replicates. Among the 34 tested Rif^R^ mutants, 13 mutants were identified positive, and 21 mutants negative for the growth of *H. bacteriophora* H06. 7 positive (LN2-R2, LN2-R6, LN2-R12, LN2-R15, LN2-R28, LN2-R31, LN2-R33) and 7 negative mutants (LN2-R3, LN2-R5, LN2-R7, LN2-R8, LN2-R11, LN2-R16, LN2-R25) were selected for further study (Table 2).

**Table 2:** *rpoB* mutations of the Rif^R^ mutants and their effect on H06 nematode growth.

Bacterial mutants	Nucleotide change	Amino acid change	Effect on the growth of H06 nematodes
LN2-R2	G436T	V146F	+
LN2-R6			
LN2-R15			
LN2-R12	C938A	A313D	+
	C1585T	R529C	
LN2-R28	C1535T	S512F	+
LN2-R33			
LN2-R31	C1537A	Q513K	+
LN2-R3	C1691T	P564L	−
LN2-R7			
LN2-R11			
LN2-R16			
LN2-R25			
LN2-R5	C521T	A174V	−
	C1691T	P564L	
LN2-R8	C826T	Q276*	−
	C1691T	P564L	
	A2475G	No change	
LN2-A	No change	No change	−
LN2-M1	No change	No change	+

+ Nematode production;

− No nematode production and nematode died after 7 days;

* stop code.

### Colonial Characterization of the Mutants and Wild-type Bacterial Strain

Colony pigmentation was determined on LB1 agar, NBTA, and MacConkey agar plates. pH-sensitive pigment production in LB1 was determined by addition of 1 M NaOH or 1 M HCl. Tests for the production of antibiotic substances were conducted as previously described [48], using *Bacillus subtilis* as test organism, and scored positive when a growth inhibition zone of >3 mm was measured around the *P. luminescens* colonies at 96 h after inoculation of the overlay culture. Bioluminescence was observed by dark-adapted eyes in a dark room. Cell morphology was observed microscopically. Catalase activity was tested by introducing 0.1 ml 3% hydrogen peroxide into the bacterial cultures and observing the release of oxygen. For all assays, both wide-type and mutant colonies were characterized on the same agar plate ad positive and negative controls. At least three plates for each medium were established.

### DNA Manipulation

Plasmid DNA preparation, extraction of genomic DNA, restriction enzyme digestions, and ligations were carried out as previously described [49]. Restriction enzymes (Promega, USA) and T4 ligase (Novagen, Germany) were used according to the manufacturer’s instructions. Plasmids were extracted from *E. coli* with QIAprep Spin Miniprep kit (Qiagen, Netherlands). When required, DNA fragments were extracted and purified from agarose gels using E.Z.N.A.™ Gel Extractio kit (Omega, USA). The genomic DNA was isolated from *P. luminescens* bacteria using E.Z.N.A.™ Bacterial DNA Kit (Omega).

### Mutation Analysis of the *rpoB* Gene from Different Strains

To examine the *rpoB* sequence from the Rif^R^ mutants and wild-type strain (LN2-W), together with a spontaneous Amp^R^ mutant LN2-A and the *namA* mutant LN2-M1 [41], the gene was amplified from the genomic DNA of different strains, by PCR with PfuUltra™ II Fusion HS DNA Polymerase (Stratagene, Germany), using the primers rpoB-BamHI-F (5′-GCTGGATCCATGGTTTACTCCTATACCGAG-3′) and rpoB-PstI-R (5′-GCACTGCAGTTATTCGTCTTCCAGCTCGATG-3′). The amplified gene was cloned into pCR4-TOPO vector (Invitrogen, USA), and transformed into *E. coli* TOP10 (Invitrogen). DNA sequencing was performed by Invitrogen Trading (Shanghai) Co. Ltd. All strains were sequenced at least twice. The sequence data of the *rpoB* gene were assembled and analyzed with DNAstar and CLUSTAL W program. The *rpoB* sequence data from wild-type strain of *P. luminescens* LN2 has been submitted to the GenBank database under accession number (JN177303).

### Allelic Exchange Mutagenesis of the *rpoB* Gene

The plasmids of pCR4-TOPO-rpoB-LW and pCR4-TOPO-rpoB-LR31 containing corresponding *rpoB* genes from LN2-W and LN2-R31, were digested with *BamH*I (GGATCC) and *Pst*I (CTGCAG), respectively. The resulting *rpoB* fragments were purified and ligated into the suicide vector pPHU281 [50] digested with *BamH*I and *Pst*I to yield plasmids pPHU281-rpoB-LW and pPHU281-rpoB-LR31. The resulting plasmids were transferred into *E. coli* S17-1 (*λpir*) [51]. Strains LN2-WΔ*rpoB*-LR31 (LN2-W containing the mutant *rpoB* allele from LN2-R31) and LN2-R31Δ*rpoB*-LW (LN2-R31containing the wild type *rpoB* allele from LN2-W) were created by allelic exchange with pPHU281-rpoB-LR31 and pPHU281-rpoB-LW, respectively, using biparental mating method. Rif^R^.Amp^R^.Tc^S^ exconjugants of LN2-WΔ*rpoB*-LR31 and Rif^S^.Amp^R^.Tc^S^ exconjugants of LN2-R31Δ*rpoB*-LW were selected on LB1 agar plates with appropriate antibiotics. The exconjugants had undergone allelic exchange and lost the wild-type or mutated copy of *rpoB* and the plasmid vehicle. The mutants were verified for the presence of the appropriate *rpoB* allele by sequencing *rpoB* gene as described above. The resulting confirmed allelic exchange mutants were determined for the nematicidal activities against the IJs of H06 as described above.

### Insecticidal Injection Assays

To check the insecticidal activity of the Rif^R^ mutants, the wild-type strain and Rif^R^ mutants of *P. luminescens* LN2 were grown overnight in LB1 broth without antibiotics, subcultured into fresh LB1 broth with 1% of bacterial culture, and incubated at 25°C for 24 h prior to injection. These cultures were washed and diluted to concentrations of 10, 100, 1000 CFU/µL in sterile phosphate buffered saline (PBS; 137 mM NaCl, 2.7 mM KCl, 10 mM NaHPO_4_, 2 mM KH_2_PO_4,_ pH 7.4). Last instar larvae of greater wax moth *Galleria mellonella* were incubated on ice for approximately 5 min. 10 µL of the diluted cultures or sterile PBS were injected into the first proleg of each of 10 insect larvae using a 30-gauge syringe (Hamilton, Reno, NV). Three replicates with 10 insect larvae per replicate were established. Insects were monitored every 6 h for 120 h post injection. Dead insects were observed to confirm the presence of red color and bioluminescence.

### Colonizations of H06 IJs by the GFP-labelled Mutants

To observe the colonization of IJ nematodes by the Rif^R^ mutant bacteria, the Rif^R^ mutant LN2-R31 positive for the growth of H06 nematodes was labeled with GFP by transposon mutagenesis of a pMini-Tn5 [52] containing an expressed *egfp* gene (pMini-lac-egfp). The pMini-lac-egfp was constructed as follows. A fragment containing an *egfp* gene and ribosome binding site (GAAGGTTTAGAC) was obtained from pUC19-egfp with primers egfp-SD2 (5′-GAAGGTTTAGACATGGGCAAAGGAGA-3′) and egfp-rev (5′-TAGCGGCCGCTTATTTGTATAGTTCATC-3′) (*Not*I). The amplified 750 bp PCR product was cloned into the pMD-18T vector (TaKaRa, Japan) and transformed into *E. coli* DH5α. Green clone on LB2 plates with ampicillin was selected to extract the plasmid pMD-egfp. A *Not*I-*Not*I fragment containing the *lac* promoter and *egfp* gene from pMD-egfp was cloned into pMD-19T Simple vector (TaKaRa) to generate pMD-lac-egfp, with primers lac-F (5′-AGCGGCCGCGAGCGCAGCGAGTCAGTGAGC-3) (*Not*I) and egfp-rev (*Not*I ). After transformed into *E. coli* DH5α, clones (Amp^R^) expressing GFP were detected using epifluorescence microscope (Nikon Eclipse 80i). To construct a transposon delivery vector pMini-lac-egfp, the *Not*I-*Not*I fragment carrying ribosome binding site, *lacZ* promoter and *egfp* gene from pMD-lac-egfp was inserted into the *Not*I site of pMini-Tn5. The ligation product was transformed into *E. coli* S17-1 (*λpir*). Clones (Km^R^) with green fluorescence were used to deliver the *egfp* gene into the chromosome of the Rif^R^ mutant LN2-R31 by diparent conjugation. Conjugants (Km^R^.Rif^R^) were selected on LB1 plates at 25°C. GFP-labeled LN2-R31 was observed to express stable green fluorescence, even in the absence of antibiotic selection.

To check the colonization of H06 IJs by the Rif^R^ mutant LN2-R31, the nematodes were cultured on sponge medium [30] inoculated with GFP-labeled LN2-R31 as described above respectively. The IJs were extracted from the sponge and observed for GFP-labeled bacteria. The IJs were also homogenized with a sterile glass homogenizer after surface sterilization with 0.1% merthiolate and 5-time rinse with sterile distilled water. The presence of the GFP-labeled bacteria retained in the IJs intestines was determined by plating dilutions of surface-sterilized and homogenized nematodes on LB1 agar plates.

### 2-DE Analysis and Protein Identification by MALDI-TOF-MS

The 48 h old bacterial cells of the wild-type strain and Rif^R^ mutants (one negative mutant LN2-R16 and three positive mutants LN2-R2, LN2-R31 and LN2-R33, for H06 growth) grown on LB1 plates at 25°C were harvested and washed three times with cold PBS by centrifugation (6000 g, 10 min, 4°C). The cell pellets were resuspended in lysis buffer (8 M urea, 0.2% w/v Bio-Lyte 3/10 Ampholyte (Bio-Rad, USA), 4% CHAPS, 65 mM DTT) containing Protease Inhibitor Cocktail (Calbiochem, Germany) and Benzonase (Novagen, Germany) and disrupted by liquid nitrogen freezing-thawing three times. Cell debris was removed by centrifugation (20000 *g*, 60 min, 4°C). The supernatant (total cell protein) was divided into aliquots and stored at −80°C until use. Protein concentrations were determined by the Bradford method using Modified Bradford Protein Assay Kit (Sangon, China).

The 2-DE was performed according to the methods described previously [53] and the manufacturer’s instruction. The first dimension (isoelectric focusing) was conducted using the IPGphor IEF system (Bio-Rad) at 20°C. For analytical gels, 350 µg protein was solubilized in 400 µL rehydration solution (8M urea, 0.2% w/v Bio-Lyte 3/10 Ampholyte, 4% CHAPS, 65 mM DTT, 0.001% w/v bromophenol blue), and loaded onto a 17 cm pH 3–10 NL IPG strip (Bio-Rad). Focusing was performed for 13 h at 50V, 1 h at 500 V, 1 h at 1000 V, and 5 h and 30 min at 8000 V (total  = 45 kVh). The IPG strips were equilibrated as previously described [53]. The second dimension was performed with 12% (w/v) SDS-polyacrylamide gels using the Protean II xi 2D Multicell system (Bio-Rad). Proteins were stained with silver nitrate, and gels were digitized using Image ScannerII (Amersham Biosciences). Digitized 2-DE gel patterns were edited and matched using the PDQUEST software package (PDI, Humington Station). Triplicate experiments were run to confirm the reproducibility of results.

Spots of interest in gels staining with silver nitrate were cut out, washed, reduced, *S*-alkylated with iodoacetamide and in-gel digested at 37°C overnight with sequencing grade porcine trypsin (Promega, USA). After extraction in extractant of 50% ACN (Fisher) and 2.5% TFA (Sigma), peptide mixtures were analyzed using a saturated solution of 5 mg/mL α-cyano-4-hydroxycinnamic acid (CHCA, Sigma) in ACN containing 0.1% TFA (Sigma) (50/50 v/v) using a 4800 Proteomics Analyzer equipped with matrix assisted laser desorption ionization time-of-flight mass spectrometry (MALDI-TOF MS) (Applied Biosystems, Framingham, MA, USA). For MS calibration, the trypsin autolysis peptides were used as internal calibrants. Monoisotopic peak masses were automatically determined within the mass range of 800–4000 Da, with a minimum S/N of 50. Five of the most intense ion signals were selected as precursors for MS/MS acquisition. Combined MS and MS/MS queries were performed with the MASCOT search engine (V2.1, Matrix Science, UK) embedded in GPS-Explorer Software (V3.6, Applied Biosystems), using the *P*. *luminescens* database (Gene DB). MASCOT protein scores (based on combined MS and MS/MS spectra) of greater than 61 were considered statistically significant (p≤0.05). The individual MS/MS spectrum with statistically significant (confidence interval >95%) best ion score (based on MS/MS spectra) were also accepted.

### Insertion-deletion Mutations of the Corresponding Genes from Differentially Expressed Proteins

Compared with the nematicidal-producing mutant LN2-R16 and wild type strain, four proteins including RplC, RhlE, NamB (a putative transport and binding protein from type III secretion system), and a hypothetical protein similar to unknown protein YggE of *E. coli* were downregulated; three proteins including DsbA, HlpA, and a hypothetical protein highly similar to unknown protein YgdH of *E. coli* were upregulated in the non-nematicidal-producing mutants (LN2-R2, LN2-R31 and LN2-R33) (Table 3–4, Figure S1, S2, S3, S4, S5). At least these 7 putative proteins were probably involved in the nematicidal activity of LN2 bacteria against H06 nematodes.

**Table 3:** Total proteins with altered level of synthesis in the nematicidal-producing and non nematicidal-producing mutants.

Spot No.	Protein Name/plu	Organism	Gene	Function	Protein PI	Protein MW	Ratio				
							W/W	R2/W	R16/W	R31/W	R33/W
**Ribosomal protein**											
6	30S ribosomal protein S8	*P. luminescens* TT01	*rpsH*	Binds directly to 16S rRNA central domain where it helps coordinate assembly of the platform of the 30S subunit	9.35	14205.6	1	0.7	0.8	0.27	1.03
15	50S ribosomal protein L17, plu4701	*P. luminescens* TT01	*rplQ*	A component of the macrolide binding site in the peptidyl transferase cente	11.04	14708.8	1	0.5	1.27	0.69	1.34
26	50S ribosomal protein L3, plu4726	*P. luminescens* TT01	*rplC*	Binds directly near the 3′ end of the 23S rRNA, where it nucleates assembly of the 50S subunit; essential for peptidyltransferase activity; mutations in this gene confer resistance to tiamulin	9.87	22328.9	1	0	0.41	0	0
44	50S ribosomal protein L9 plu4570	*P. luminescens* TT01	*rplI*	In *E. coli* this protein is wrapped around the base of the L1 stalk	6.13	15872.5	1	2.38	2.23	0.92	1.37
45	50S ribosomal protein, L9 plu4570	*P. luminescens* TT01	*rplI*	In *E. coli* this protein is wrapped around the base of the L1 stalk	6.13	15872.5	1	1.82	3.29	0.51	1.23
**Adaptations conditions**											
7	Hypothetical protein, plu2032	*P. luminescens* TT01		Similar to Unknown protein YbdQ of *E. coli*; Similar to universal stress protein (pfam00582: Usp)	6.19	15907.4	1	3.79	2.9	1.46	4.83
9	Hypothetical protein, plu2030	*P. luminescens* TT01		Similar to Unknown protein YbdQ of *E. coli*, Similar to universal stress protein (pfam00582: Usp)	5.76	15283.2	1	1.71	1.21	0.2	0.45
48	Alkyl hydroperoxide reductase, small subunit (antioxidant), plu3907	*P. luminescens* TT01	*ahpC*	Alkyl hydroperoxide reductase, small subunit (antioxidant)	5.98	22259.3	1	0.8	0.82	0.9	0.47
**Secondary metabolites**											
12	Crystalline inclusion protein CipB	*P. luminescens* TT01	*cipB*	Unknown, similar to crystalline inclusion protein type II	6.08	11281.6	1	0.12	0	0.51	0
25	Crystalline inclusion protein CipA, plu1576	*P. luminescens* TT01	*cipA*	Crystalline inclusion protein CipA	6.06	11710.9	1	0	0.08	0.74	0
32	Unknown	*P. luminescen*		Similar to hemolysin from *Fusobacterium nucleatum* clinical isolate found in GenBank, Accession Number AF525507	5.61	39995.2	1	0.9	1.7	0.44	0.8
62	Hypothetical protein, plu4211	*P. luminescens* TT01		Highly similar to Hcp protein	6.29	18482.5	1	11.43	14.91	19.98	3.24
**Metabolism of amino acids and related molecules**											
30	Ethanolamine ammonia-lyase small subunit, plu2971	*P. luminescens* TT01	*eutC*	Catalyzes the formation of acetaldehyde from ethanolamine	6.37	31188.4	1	4.17	0.5	1.1	1.2
36	Tryptophan synthase subunit beta, plu2466	*P. luminescens* TT01	*trpB*	Catalyzes the formation of L-tryptophan from L-serine and 1-(indol-3-yl)glycerol 3-phosphate	6.2	43098	1	1.22	0.25	0.41	0.58
46	Serine/arginine repetitive matrix protein 2		*Srrm2*		12.02	294500.8	1	0.72	1.7	0.89	0.9
50	2,3-dihydroxy-2,3-dihy drophenylpropionate dehydrogenase, plu2207	*P. luminescens* TT01	*hcaB*	Converts cis-3-(3-carboxyethyl) -3,5-cyclohexadiene-1,2-diol (PP-dihydrodiol) into 3-(2,3-dihydroxylphenyl) propionate	5.43	29519.1	1	0.37	0.3	0.49	0.9
51	Hypothetical protein, plu4676	*P. luminescens* TT01		Similar to 3-oxoacyl-[acyl-carrier-protein] synthase II (beta-ketoacyl-ACP synthase II) (KAS II)	5.58	45407.3	1	3.29	3.0	0.01	<0.01
52	Serine hydroxymethyltransferase, plu3291	*P. luminescens* TT01	*glyA*	Catalyzes the reaction of glycine with 5,10-methylenetetrahydrofolate to form L-serine and tetrahydrofolate”	5.92	45229.8	1	3.74	4.55	2.93	5.6
53	Urease accessory protein, plu2176	*P. luminescens* TT01	*ureG*	Urease accessory protein	5.04	22868.1	1	4.42	2.98	5.17	6.66
54	Hypothetical protein, plu2040	*P. luminescens* TT01		Similar to vibrio bactin utilization protein ViuB	5.51	31363	1	2.36	3.61	1.83	6.57
55	3-oxoacyl-(acyl carrier protein) synthase III, plu2835	*P. luminescens* TT01	*fabH*	FabH; beta-ketoacyl-acyl carrier protein synthase III; catalyzes the condensation of acetyl-CoA with malonyl-ACP to initiate cycles of fatty acid elongation; differs from 3-oxoacyl-(acyl carrier protein) synthase I and II in that it utilizes CoA thioesters as primers rather than acyl-ACPs”	5.33	34189.6	1	5.12	6.14	3.85	6.77
**Nucleosides and nucleotides biosynthesis and metabolism**											
1	Hypothetical protein, plu3994	*P. luminescens* TT01		Similar to putative membrane protein YqjD (carboxyl transferase) of *E. coli*	7.93	11042	1	4.03	0.57	2.91	1.2
3	IS630 family transposase, plu0720	*P. luminescens* TT01	*ISPlu3Y*	Transposase, IS630 family	9.35	39771.5	1	0.38	0.17	0.02	0.11
47	IS630 family transposase, plu0468	*P. luminescens* TT01	*ISPlu10J*	Transposase	9.59	39687.9	1	0.85	0.6	0.71	0.25
4	ATP-dependent RNA helicase RhlE, plu1511	*P. luminescens* TT01	*rhlE*	This helicase is not essential cell growth	10.01	48258.8	1	0.05	0.52	0.04	0.02
19	ATP-dependent RNA helicase RhlE, plu1511	*P. luminescens* TT01	*rhlE*	This helicase is not essential cell growth	10.01	48258.8	1	0	1.43	0.2	0
18	Nucleoside diphosphate kinase, plu1372	*P. luminescens* TT01	*ndk*	Catalyzes the formation of nucleoside triphosphate from ATP and nucleoside diphosphate	5.35	15591.8	1	1.43	1.96	2.48	2.53
57	Uracil phosphoribosyl transferase, plu2759	*P. luminescens* TT01	*upp*	Catalyzes the formation of uracil and 5-phospho-alpha-D-ribosy 1-diphosphate from UMP and diphosphate	5.46	22489	1	1.59	2.81	1.83	4.95
58	Reverse gyrase	*Leptospirillum* sp. Group II UBA			9.31	56458	1	0	4.93	6.32	0.41
**Cell wall/membrane biogenesis**											
8	Karst CG12008-PA, isoform A	*Drosophila melanogaster*	*kst*	Kst-PA; spectrin beta-heavy chain; beta-H spectrin;	5.93	471351.1	1	2.20	1.8	1.33	1.16
43	hypothetical protein, plu3994	*P. luminescens* TT01		Similar to putative membrane protein YqjD of *E. coli*	7.93	11042	1	0.21	0.05	0.1	0.08
5	Periplasmic chaperone, plu0681	*P. luminescens* TT01	*hlpA*	Histone-like protein HLP-1 precursor	9.43	18476.8	1	2.63	0.81	2.11	2.64
**Transport and binding proteins**											
10	Unknown	*P. luminescens* W14		From (type III secretion system, partial sequence) GI:27550090	6.1	16883.6	1	0	1.53	0	0
64	Unknown	*P. luminescens* w14		From (type III secretion system, partial sequence) GI:27550090	6.1	16883.6	1	0.1	0.91	0.11	0.15
65	Unknown	*P. luminescens* w14		From (type III secretion system, partial sequence) GI:27550090	6.1	16883.6	1	0.11	0.93	0.11	0.16
11	Hypothetical protein, plu1886	*P. luminescens* TT01		Hypothetical transmembrane protein	5.88	15588.6	1	1.53	2.83	1.57	0.43
13	Macrolide transporter subunit MacA, plu1590	*P. luminescens* TT01	*macA*	Probable macrolide-specific ABC transporter; confers macrolide resistance via active drug efflux	6.95	40743.2	1	0	0	0	0
17	Sec-independent protein translocase protein, plu4410	*P. luminescens* TT01	*tatA*	Sec-independent protein translocase protein	6.18	9289.9	1	0.76	0.81	0.72	0.23
42	Na-binding protein HU-alpha (NS2) (HU-2), plu0492	*P. luminescens* TT01	*dbhA*	Na-binding protein HU-alpha (NS2) (HU-2)	9.1	9407	1	2.1	2.3	0.59	0.99
**Information and regulatory pathways**											
14	DNA-binding transcriptional regulator HexR, plu2121	*P. luminescens* TT01	*hexR*	Represses the expression of the *zwf*, *eda*, *glp* and *gap*	6.97	31694.5	1	2.2	1.96	1.92	0.91
16	DnaK transcriptional regulator DksA, plu0876	*P. luminescens* TT01	*dksA*	DnaK transcriptional regulator DksA	5.04	17415.8	1	0	0.05	0.65	0
20	Nucleotide-binding protein, plu3881	*P. luminescens* TT01		Similar to Unknown protein YajQ of *E. coli*	5.77	18311.4	1	1.47	0.89	1.48	1.09
22	Transcriptional repressor MprA, plu1277	*P. luminescens* TT01	*mprA*	DNA-binding transcriptional repressor of microcin B17 synthesis and multidrug efflux; negative regulator of the multidrug operon *emrAB*”	7.01	20365.5	1	1.67	1.18	1.38	1.09
27	Hypothetical protein, plu0318	*P. luminescens* TT01		Similar to AidA protein of *Ralstonia solanacearum*	5.7	22068.3	1	1.03	1.68	0.86	0.87
49	Periplasmic protein disulfide isomerase I, plu0381	*P. luminescens* TT01	*dsbA*	Disulfide interchange protein DsbA precursor	7.7	22954.8	1	3.8	1.57	3.49	3.62
37	Protease precursor DegQ, plu4018	*P. luminescens* TT01	*degQ*	Protease precursor DegQ	9.12	48028.9	1	0	0	0	0
38	Protease precursor DegQ, plu4018	*P. luminescens* TT01	*degQ*	Protease precursor DegQ	9.12	48028.9	1	0	0	0	0
**Energy production and conversion**											
24	GD22749	*Drosophilia simulans*	*Dism\GD22749*	Chromosome segregation ATPases [Cell division and chromosome partitioning]; COG1196	5.23	76326.8	1	1.39	0	<0.01	<0.01
23	Hypothetical protein, plu2075	*P. luminescens* TT01		Similar to 3-oxoacyl-[acyl-carrier protein] reductase	8.55	25032.6	1	0.12	0.31	0.45	0.33
35	WblA protein, plu4796	*P. luminescens* TT01	*wblA*	Probable lipopolysaccharide biosynthesis protein; Similar to putative UDP-glucose/GDP-mannose dehydrogenase	5.86	48484	1	2.1	0	2.07	0.05
40	Catalase, plu3068	*P. luminescens* TT01	*katE*	Catalase	6.92	55509.5	1	0	0	0	0
41	Catalase plu3068	*P. luminescens* TT01	*katE*	Catalase	6.92	55509.5	1	0	0	0	0
56	Phosphoglycero mutase, plu1471	*P. luminescens* TT01	*gpmA*	Catalyzes the interconversion of 2-phosphoglycerate to 3-phosphoglycerate	5.62	28396.7	1	1.64	2.88	2.02	3.5
**Phage-related proteins**											
28	Hypothetical protein, plu3012	*P. luminescens* TT01		Probable phage protein; Similar to tail fiber assembly protein from bacteriophage	4.67	21467.8	1	0.68	0.88	2.36	0.44
29	Hypothetical protein, plu2035	*P. luminescens* TT01		Some similarities with putative tail fiber protein of prophage	4.23	23641.5	1	0	3	0.42	0.88
39	Hypothetical protein, plu3803	*P. luminescens* TT01		Some similarities with prophage tail fiber protein	6.37	66850.4	1	0.23	0.5	0.1	0
60	Hypothetical protein, plu3032	*P. luminescens* TT01		Putative bacteriophage protein; Some similarities with Unknown protein of *Photorhabdus*	6.08	22021.6	1	1.93	2.55	2.14	4.66
63	Hypothetical protein, plu3012	*P. luminescens* TT01		Probable phage protein; Similar to tail fiber assembly protein from bacteriophage	4.67	21467.8	1	1.66	1.72	5.27	0.48
**Flagellin**											
31	Flagellin, plu1954	*P. luminescens* TT01	*fliC*	Structural flagella protein	5.19	38183.6	1	0	0.1	0.87	0
**Post-translational modification**											
61	PTS system, N-acetyl- galactosamine-specific IIB component 2 (EIIB-AGA’) (N-acetyl- galactosamine-perme, plu0835	*P. luminescens* TT01	*agaV*	Probable PTS system; Highly similar to PTS system, cytoplasmic, N-acetylgalactosamine-specific	6.51	17774.4	1	3.87	2.18	4.31	1.5
**Uknown**											
2	Hypothetical protein, plu0661	*P. luminescens* TT01		Highly similar to Unknown protein YgdH of *E. coli*	5.95	51257.2	1	2.74	0.81	2.51	1.76
21	Unknown	Fanconi anemia group D1 protein	*LOC725687*	Similar to Breast cancer type 2 susceptibility protein	8.75	82829.5	1	0.05	0	0	0
33	Hypothetical protein, plu3611	*P. luminescens* TT01		Similar to Unknown protein YggE of *E. coli*	5.97	25893.5	1	0	2.33	0.2	0.4
34	Hypothetical protein MHP7448_0445	*Mycoplasma hyopneumoniae* 7448		GI:72080777	9.17	275940.9	1	0.39	0.19	0.1	0.05
59	Hypothetical protein UM02446.1	*Ustilago maydis* 521		GI:124514614	8.96	130494.3	1	11.46	2.78	6.42	6.6

**Table 4:** Total proteins with altered level of synthesis in the nematicidal-producing and non nematicidal-producing mutants.

Spot No.	Protein Name/plu	Organism	Gene	Function	Ratio				
					nematicidal-producing strains		non nematicidal-producing strains		
					W/W	R16/W	R2/W	R31/W	R33/W
26	50S ribosomal protein L3, plu4726	*P. luminescens* TT01	*rplC*	Binds directly near the 3′ end of the 23S rRNA, where it nucleates assembly of the 50S subunit; essential for peptidyltransferase activity; mutations in this gene confer resistance to tiamulin	1	0.41	0	0	0
4	ATP-dependent RNA helicase RhlE, plu1511	*P. luminescens* TT01	*rhlE*	This helicase is not essential cell growth	1	0.52	0.05	0.04	0.02
19	ATP-dependent RNA helicase RhlE, plu1511	*P. luminescens* TT01	*rhlE*	This helicase is not essential cell growth	1	1.43	0	0.2	0
5	Periplasmic chaperone, plu0681	*P. luminescens* TT01	*hlpA*	Histone-like protein HLP-1 precursor	1	0.81	2.63	2.11	2.64
10	Unknown	*P. luminescens* W14		From (type III secretion system, partial sequence) GI:27550090	1	1.53	0	0	0
64	Unknown	*P. luminescens* w14		From (type III secretion system, partial sequence) GI:27550090	1	0.91	0.1	0.11	0.15
65	Unknown	*P. luminescens* w14		From (type III secretion system, partial sequence) GI:27550090	1	0.93	0.11	0.11	0.16
49	Periplasmic protein disulfide isomerase I, plu0381	*P. luminescens* TT01	*dsbA*	Disulfide interchange protein DsbA precursor	1	1.57	3.8	3.49	3.62
2	Hypothetical protein, plu0661	*P. luminescens* TT01		Highly similar to Unknown protein YgdH of *E. coli*	1	0.81	2.74	2.51	1.76
33	Hypothetical protein, plu3611	*P. luminescens* TT01		Similar to Unknown protein YggE of *E. coli*	1	2.33	0	0.2	0.4

To confirm these results, the downregulated *rhlE* and *namB*, and upregualted *dsbA* (GenBank accession number JX274431, JX274430 and JX274432 respectively) from the differentially expressed proteins were selected for construction of insertion-deletion mutations to determine the effects of the knock-out genes on the nematicial activity. Three *P. luminescens* LN2 mutants termed as LN2Δ*rhlE*, LN2Δ*namB* and LN2Δ*dsbA* were created. Insertion-deletion mutations in these three genes were constructed using fusion PCR strategy as previously described [54]. For each gene, three fragments F1 (the upstream of the target gene), *camR* (Chloramphenicol cassette) and F2 (the downstream of the target gene) were generated using primer pairs of P1 and P2, P3 and P4, and P5 and P6 (Table S1), respectively. The *camR* gene was amplified from the plasmid pSZ21 [55] and the F1 and F2 gene fragments were amplified from *P. luminescens* LN2 genomic DNA. Approximately equal amounts of the three purified fragments F1, *camR* and F2 were mixed, and used as a template to generate a new DNA fragment by a second PCR performed with the primers P1 and P6. Three resulting fragments, which corresponded to *rhlE*::Cm, *namB*::Cm and *dsbA*::Cm, respectively, were separately cloned into a pMD-19T Simple vector. Then the resulting plasmids of pMD-*rhlE*::Cm, pMD-*namB*::Cm and pMD-*dsbA*::Cm were separately ligated to the same enzyme digested suicide vector pKNG101 [56] to generate pKNG101-*rhlE*::Cm, pKNG101- *namB*::Cm and pKNG101-*dsbA*::Cm. *P. luminescens* LN2 mutants termed as LN2Δ*rhlE*, LN2Δ*namB* and LN2Δ*dsbA* were created by allelic exchange with pKNG101-*rhlE*::Cm, pKNG101- *namB*::Cm and pKNG101-*dsbA*::Cm, respectively, as previously described [54]. The phenotypic characterization, *rpoB* sequence and effects on nematicidal activity of three resulting mutants were determined as described above.

## Results

### Isolation and Characterization of the Rif^R^ Mutants of *P. luminescens* LN2

Several hundreds of the Rif^R^ mutants of *P. luminescens* LN2 were isolated and 34 mutants were randomly selected for further study. The wild type strain and the selected mutants showed the typical characteristics of phase one bacteria as described: uptake of dye from NBTA and MacConkey agar, production of pH-sensitive pigments, occurrence of inclusion bodies, antibiotic activity, and bioluminescence.

### The Effects of the Rif^R^ Mutants on the Growth of H06 Nematodes

13 of 34 Rif^R^ *P. luminescens* LN2 mutants were able to support the growth of H06 IJs, with hermaphrodites containing living juveniles inside and outside after 12 days on the agar plates, while 21 of them were negative for the growth of H06 nematodes. On the bacterial lawns with those mutants or the wild-type, which did not support the nematode production, all the nematodes did not grow beyond adults and died after 7 days.

### The Mutation Loci of *rpoB* Gene in the Rif^R^ Mutants

7 positive mutants (LN2-R2, LN2-R6, LN2-R12, LN2-R15, LN2-R28, LN2-R31 and LN2-R33) and 7 negative mutants (LN2-R3, LN2-R5, LN2-R7, LN2-R8, LN2-R11, LN2-R16 and LN2-R25) for H06 nematode growth (Table 2) were randomly selected for *rpoB* gene sequencing. The entire *rpoB* sequences of 14 selected Rif^R^ mutants, wild type strain, LN2-A and LN2-M1 were sequenced at least twice. The *rpoB* gene from all colonies was 4029 bp in length, the same to that of *P. luminescens* subsp. laumondii TT01 [57]. The identity of *rpoB* genes between the wild-type strain of LN2 and TT01 was 96.13%. All of the 14 Rif^R^ mutants carried mutations in the *rpoB* gene. 10 mutants showed a single nucleotide mutation resulting in an amino acid substitution, and 2 mutants presented two nucleotide mutations resulting in two amino acid substitutions, but only one mutant displayed three nucleotide mutations resulting in two amino acid substitutions (Table 2). No mutation was observed in the *rpoB* gene of Amp^R^ mutant LN2-A and *namA* mutant LN2-M1.

The *rpoB* (P564L) mutation was found in all 7 mutants which produced nematicidal activity against H06 nematodes, but not in the mutants which supported H06 nematode production. While the single mutations of V146F, S512F, Q513K and double mutations of A313D and R529C were detected respectively in the mutants which supported H06 nematode production (Table 2). The single and double mutations resulted in loss of nematicidal activity against H06 nematodes and ability to supported H06 nematode production.

### Allelic Exchange Assays

The recombinant LN2-WΔ*rpoB*-LR31 (LN2-W strain containing the mutant *rpoB* allele from LN2-R31) and the LN2-R31Δ*rpoB*-LW (LN2-R31 containing the wild type *rpoB* allele from LN2-W) were selected on Am^R^ Rif^R^ Tc^s^ and Am^R^ Rif^S^ Tc^s^ LB1 agar plates, respectively.

Successful homologous recombination of *rpoB* gene in the recipient strains was verified by randomly selecting three colonies from each recipient and checking their *rpoB* gene sequences by PCR and DNA sequencing. The sequences of *rpoB* gene from three colonies of each recipient were 100% identical.

The recombinant LN2-WΔ*rpoB*-LR31 showed Rif resistance and lost the nematicidal activity against H06 IJs, while LN2-R31Δ*rpoB*-LW was sensitive to Rif and restored the nematicidal activity. These results clearly indicated that *rpoB* mutation was responsible for the Rif-resistance and the absence of nematicidal activity of LN2-R31.

### Insecticidal Activity

The Rif^R^ mutants, including nematicidal-producing LN2-R16 and non nematicidal-producing LN2-R2, LN2-R31 and LN2-R33, together with wild-type of *P. luminescens* LN2 caused 100% mortality of *G. mellonella* at the concentrations of 1000 CFU/µL after 24 h, 100 CFU/µL after 30 h, and 10 CFU/µL after 36 h. No insect mortality was recorded in the control after 120 h. It appeared that the mutant bacteria also displayed insecticidal activity against *G. mellonella* larvae.

### IJ Colonization of the GFP-labelled Mutants

No H06 IJs from the culture with GFP-labeled LN2-R31 mutant contained GFP-labeled bacteria in their intestines. No GFP-labeled bacteria were also observed from the mechanically disrupted H06 IJs. However, the IJs of *H. indica* LN2 from GFP-labeled LN2-R31 mutant contained GFP-labeled bacteria in their intestines. Bacterial colonization of the intestines of IJs is an important process in the nematode-bacterium symbiosis. The present result demonstrated that the mutation of *rpoB* gene restored the nutrient suitability of the LN2 bacteria for the reproduction of H06 nematodes by silencing the nematicidal activity of the bacteria, but did not establish the environment for bacterial colonization of the IJs.

### The Proteomic Analysis of the Mutants and Wild-type

The effects of *rpoB* mutations on the nematicidal activity of LN2 bacteria were further investigated by identifying the differentially expressed proteins by 2-DE. The parental and selected *rpoB* mutant strains grown on LB1 agar plates were collected after 48 h. Cells were disrupted and whole cell proteins were separated on 2-DE gels spanning the pH 3–10, silver stained, and analyzed by MS. Protein levels were expressed as percentage volume, which corresponds to the percentage ratio between the volume of a single spot and the total volume of all spots present in a gel. The mean values of spot intensity were calculated using at least three gels. Spots showing more than 15% variation were not considered (Student’s test, with 7 degrees of freedom, p<0.05). Little deviation was observed in the patterns on replica gels.

Approximately 900 spots were revealed on the silver-stained 2-DE patterns of the whole cell proteins from wild type strain LN2-W and the mutant strains of LN2-R16, LN2-R2, LN2-R31 and LN2-R33 (Figure S1, S2, S3, S4, S5). Protein spots were distributed over the 3–10 pH range, with most spots in the 4–7 pH range.

Major differences were detected from different *rpoB* mutants (Figure S1, S2, S3, S4, S5, Table 3). Comparing to the wild type strain, 19, 12 and 13 spots were differentially upregulated, downregulated or missing, respectively, by a factor of at least two in the *rpoB* mutant LN2-R2; 19, 12 and 9 spots in the LN2-R16; 17, 19 and 8 spots in the LN2-R31; and 13, 19 and 14 spots in the LN2-R33.

The spots with intensity changes by a factor of at least two were selected for MALDI-TOF-MS analysis (Table 3), using NCBI website and the PhotoList database (http://genolist.pasteur.fr/PhotoList/).

The proteins identified could be classified into thirteen categories based on functions: (1) ribosomal protein, (2) adaptation conditions, (3) secondary metabolities, (4) metabolisim of amino acids and related molecules, (5) nucleosides and nucleotides biosynthesis and metabolism, (6) cell wall/membrane biogenesis, (7) transport and binding proteins, (8) information and regulation pathways, (9) energy production and conversion, (10) phage-related proteins, (11) flagellin, (12) post-translational modification, and (13) other functions and unknown. A list of the proteins affected by *rpoB* mutation was shown in Table 3.

Proteomic analysis revealed major difference between wild-type strain and Rif^R^ mutants, and between nematicidal-producing and non nematicidal-producing mutants. In all the analyzed *rpoB* mutants, 15 putative proteins (YbdQ, Hcp, GlyA, UreG, ViuB, FabH, Ndk, Upp, Kst, MprA, DsbA, GpmA, AgaV, one bacteriophage protein and one unknown protein) were upregulated, and 11 (AhpC, CipA, cipB, HcaB, ISPPlu3Y, ISPlu10J, YqjD, TatA, DksA, FliC, and three hypothetical proteins) were downregulated. In particular, the following putative proteins were not detected from all the analyzed *rpoB* mutants: MacA (probable macrolide-specific ABC transporter, spot 13); DegQ (protease precursor, spot 37, 38); and KatE (catalase, spot 40, 41). Interestingly, an unknown function protein Brca2 (similar to breast cancer type 2 susceptibility protein, spot 21) was not present in the mutants, but present in the wild type strain. It appeared that the absence of these proteins was due to the *rpoB* mutation rather than the antibiotic pressure, because they were absent also from a *namA* disruption mutant of LN2 [41] without *rpoB* mutation in the culture without any rifampin (unpublished data).

Compared with the nematicidal-producing mutant LN2-R16 and wild type strain, four proteins in the non-nematicidal-producing mutants (LN2-R2, LN2-R31 and LN2-R33) were downregulated at least a 2-fold difference in expression, including RplC (putative ribosomal protein, spot 26), RhlE (putative nucleosides and nucleotides biosynthesis and metabolism protein, spot 4, 19), NamB (a putative transport and binding protein from type III secretion system, part of T3SS, spot 10, 64, 65) and a hypothetical protein (similar to unknown protein YggE of *E. coli*, spot 33); three proteins including DsbA (periplasmic protein disulfide isomerase I involved in information and regulatory pathways, spot 49), HlpA (periplasmic chaperone involved in cell wall/membrane biogenesis, spot 5), and a hypothetical protein(highly similar to unknown protein YgdH of *E. coli*, spot 2) were upregulated in the non-nematicidal-producing mutants(LN2-R2, LN2-R31 and LN2-R33) (Table 4, Figure S1, S2, S3, S4, S5). It was suggested that at least these 7 proteins were involved in the nematicidal activity of LN2 bacteria against H06 nematodes.

### Genetic Confirmation of Differentially Expressed Proteins

LN2Δ*rhlE*, LN2Δ*namB* and LN2Δ*dsbA* mutants showed the typical characteristics of phase one bacteria as the wild type strain. No mutation in *rpoB* gene was observed in these mutants. LN2Δ*rhlE* and LN2Δ*namB* mutants were able to support the growth of H06 IJs, with hermaphrodites containing living juveniles inside and outside after 12 days on the agar plates, while LN2Δ*dsbA* mutant was negative for the growth of H06 nematodes as the wild type strain. The results confirmed the involvement of these selected genes in the nematicidal activity against H06 nematodes.

## Discussion

In this study, a similar mechanism determining Rif-resistance in *E. coli* and *M. tuberculosis* [3], [11], [58] was verified in *Photorhabdus* bacteria. Surprisingly, the Rif^R^ mutants influenced the nematicidal activity of *P. luminescens* LN2 bacteria against a different nematode, *H. bacteriophora* H06. Furthermore, some but not all *rpoB* mutants of LN2 bacteria lost nematicidal activity against H06 IJs. The *rpoB* mutation was demonstrated to be responsible for the Rif-resistance and the effect on the nematicidal activity in the Rif^R^ mutants of LN2. There are fundamental connections between rifampin resistance, RNA polymerase structure and function and global gene expression in the literatures. Rif mutations in *E. coli* affected a wide variety of phenotypes, including altered growth properties and stimulated secondary metabolism [59]. A novel *rpoB* mutation in *B. subtilis* showed a unique spectrum of effects on growth and various developmental events [60]. An *rpoB* mutation in *Streptomyces lividans* activated antibiotic production and reduced growth rate [61]. A spontaneous Rif^R^ mutation isolated from *Saccharopolyspora erythraea* stimulated bacterial secondary metabolism and was severely impaired in erythromycin production [62]. To the best of our knowledge, this is the first report that *rpoB* mutations influenced the nematicidal activitity of a nematode symbiont on a non-cognate nematode partner. The symbiosis between the entomopathogenic nematodes and their associated bacteria will be also influenced by the *rpoB* mutations. However, how *rpoB* mutations affect this nematicidal activity needs to be further explored.

The mutants exhibited several phenotypes of phase one variant as previously described [47], e.g. absorption of the dye from NBTA and MacConkey agar, production of bioluminescence and occurrence of crystalline inclusion proteins in the cells. It was reported that the nematicidal activity occurred only in phase one of *P. luminescens* LN2 [31]. Apparently, the loss of nematicidal activity in the LN2 mutants against H06 nematodes was not the result of a typical phase variation. The physiological status of symbiotic *Photorhabdus* and *Xenorhabdus* bacteria (such as phase variation, mutants) may influence their fitness for nematode production. As *rpoB* mutations were associated with the nematode growth, screening of *rpoB* mutants of symbiotic bacteria of entomopathogenic nematodes may provide a way to select beneficial *rpoB* mutants by Rif for effective mass production of the nematodes.

One of the important characters in *Photorhabdus* bacteria is their insecticidal activities towards different insects [32]–[33], [35]. The present result indicated that the *rpoB* mutations did not change the expression of the toxin genes, at least in the tested mutants, for the mutants also displayed the insecticidal activity against *G. mellonella* larvae.

Different *rpoB* mutations were associated with their ability to support H06 nematode production. However, the P564L mutation was not associated with the loss of nematicidal activity. The reasons why the mutations affect the physiology and metabolism of the bacterial mutants are not known. The RNA polymerase complex may contact every promoter in the genome, thus any change in critical portions of the enzyme can lead to global changes in gene transcription. Mutations within the Rif binding pocket of *rpoB* gene may alter the structure of RNA polymerase and hence its regulated interaction with specific promoters, and hence physiology and metabolism [63].

Proteomic analysis revealed at least 7 putative proteins including DsbA, RhlE, NamB (a protein from T3SS), HlpA, RplC and 2 hypothetical proteins YggE and YgdH might be involved in the nematicidal activity. All these proteins may play different roles in different organisms (64–71). In the present study, it was hard to establish the functional relationship among these proteins in the nematicidal activity of LN2 bacteria. However, the insertion-deletion method confirmed the involvement of the selected corresponding genes (such as *rhlE*, *namB* and *dsbA*) from the differentially expressed proteins in the nematicidal activity against H06 nematodes. It seems that a big network system is involved in this nematicidal acitivity. Further work is needed to explore this system to understand the molecular mechanism on the trans-specific nematicidal activity of incompatible symbionts.

## Supporting Information

Figure S1 **2-DE map of total cell proteins from** ***P. luminescens*** **LN2 wild type strain.** A representative gel shows the identified differentially expressed protein spots. 350 µg of total cell proteins was loaded onto a 17 cm pH 3–10 NL IPG strip, separated in the second dimension by SDS-polyacrylamide gel electrophoresis on a 12% gel and stained with silver nitrate.
(TIF)

Figure S2 **2-DE map of total cell proteins from** ***P. luminescens*** **LN2 Rif^R^ mutant LN2-R2.** A representative gel shows the identified differentially expressed protein spots. 350 µg of total cell proteins was loaded onto a 17 cm pH 3–10 NL IPG strip, separated in the second dimension by SDS-polyacrylamide gel electrophoresis on a 12% gel and stained with silver nitrate.
(TIF)

Figure S3 **2-DE map of total cell proteins from** ***P. luminescens*** **LN2 Rif^R^ mutant LN2-R16.** A representative gel shows the identified differentially expressed protein spots. 350 µg of total cell proteins was loaded onto a 17 cm pH 3–10 NL IPG strip, separated in the second dimension by SDS-polyacrylamide gel electrophoresis on a 12% gel and stained with silver nitrate.
(TIF)

Figure S4 **2-DE map of total cell proteins from** ***P. luminescens*** **LN2 Rif^R^ mutant LN2-R31.** A representative gel shows the identified differentially expressed protein spots. 350 µg of total cell proteins was loaded onto a 17 cm pH 3–10 NL IPG strip, separated in the second dimension by SDS-polyacrylamide gel electrophoresis on a 12% gel and stained with silver nitrate.
(TIF)

Figure S5 **2-DE map of total cell proteins from** ***P. luminescens*** **LN2 Rif^R^ mutant LN2-R33.** A representative gel shows the identified differentially expressed protein spots. 350 µg of total cell proteins was loaded onto a 17 cm pH 3–10 NL IPG strip, separated in the second dimension by SDS-polyacrylamide gel electrophoresis on a 12% gel and stained with silver nitrate.
(TIF)

Table S1 **Oligonucleotide sequences used to generate** ***Photorhabdus luminescens*** **LN2 mutant constructs in this study.**
(DOC)
