# Supplementary figures and images for: Genetic and Proteomic Characterization of rpoB Mutations and Their Effect on Nematicidal Activity in Photorhabdus luminescens LN2
Source: PLoS One. 2012 Aug 17;7(8):e43114. doi: 10.1371/journal.pone.0043114 (PMC3422287; doi:10.1371/journal.pone.0043114)

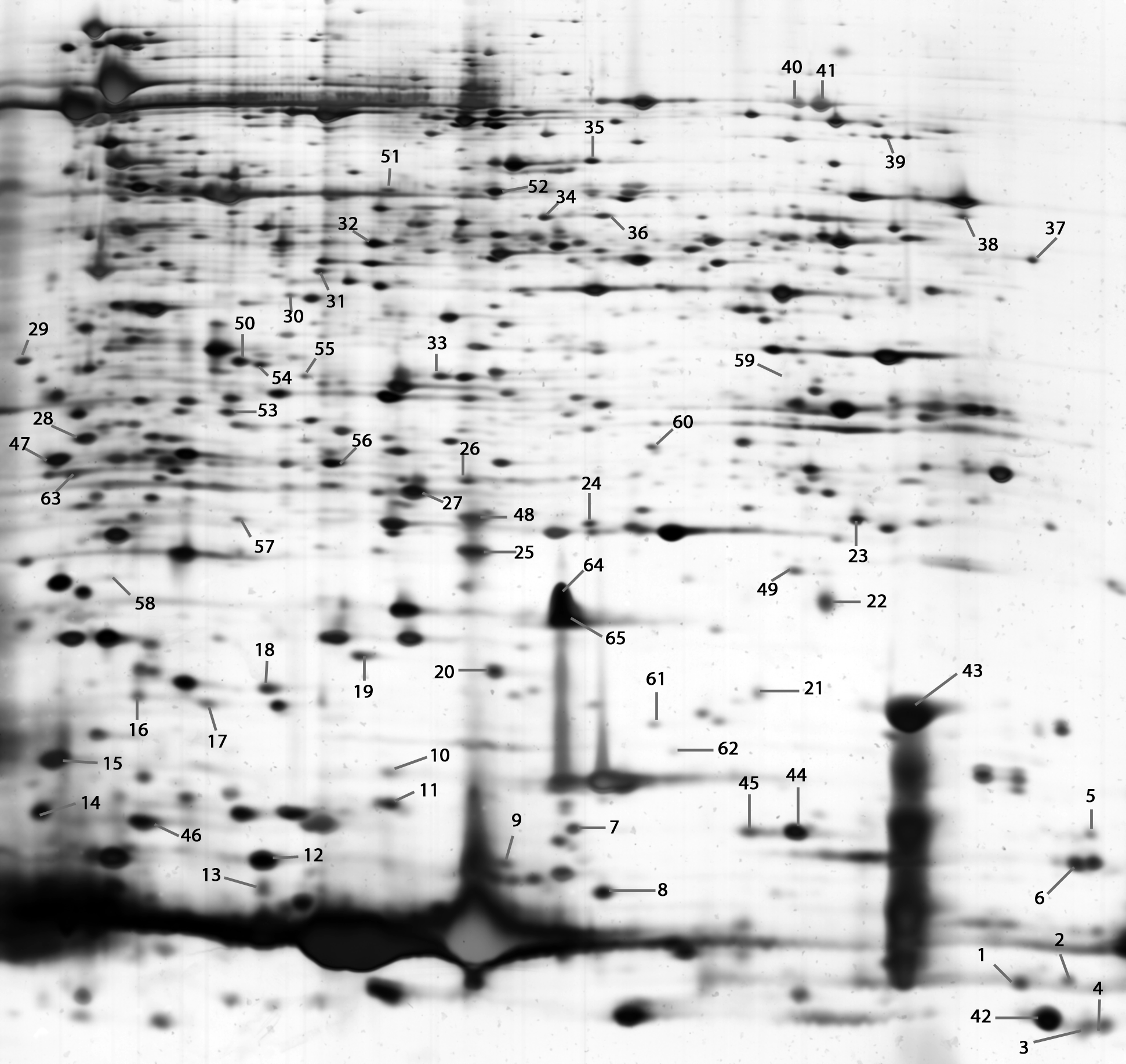

Supplement: Figure S1 — 2-DE map of total cell proteins from P. luminescens LN2 wild type strain. A representative gel shows the identified differentially expressed protein spots. 350 µg of total cell proteins was loaded onto a 17 cm pH 3–10 NL IPG strip, separated in the second dimension by SDS-polyacrylamide gel electrophoresis on a 12% gel and stained with silver nitrate. (TIF) [file pone.0043114.s001.tif]

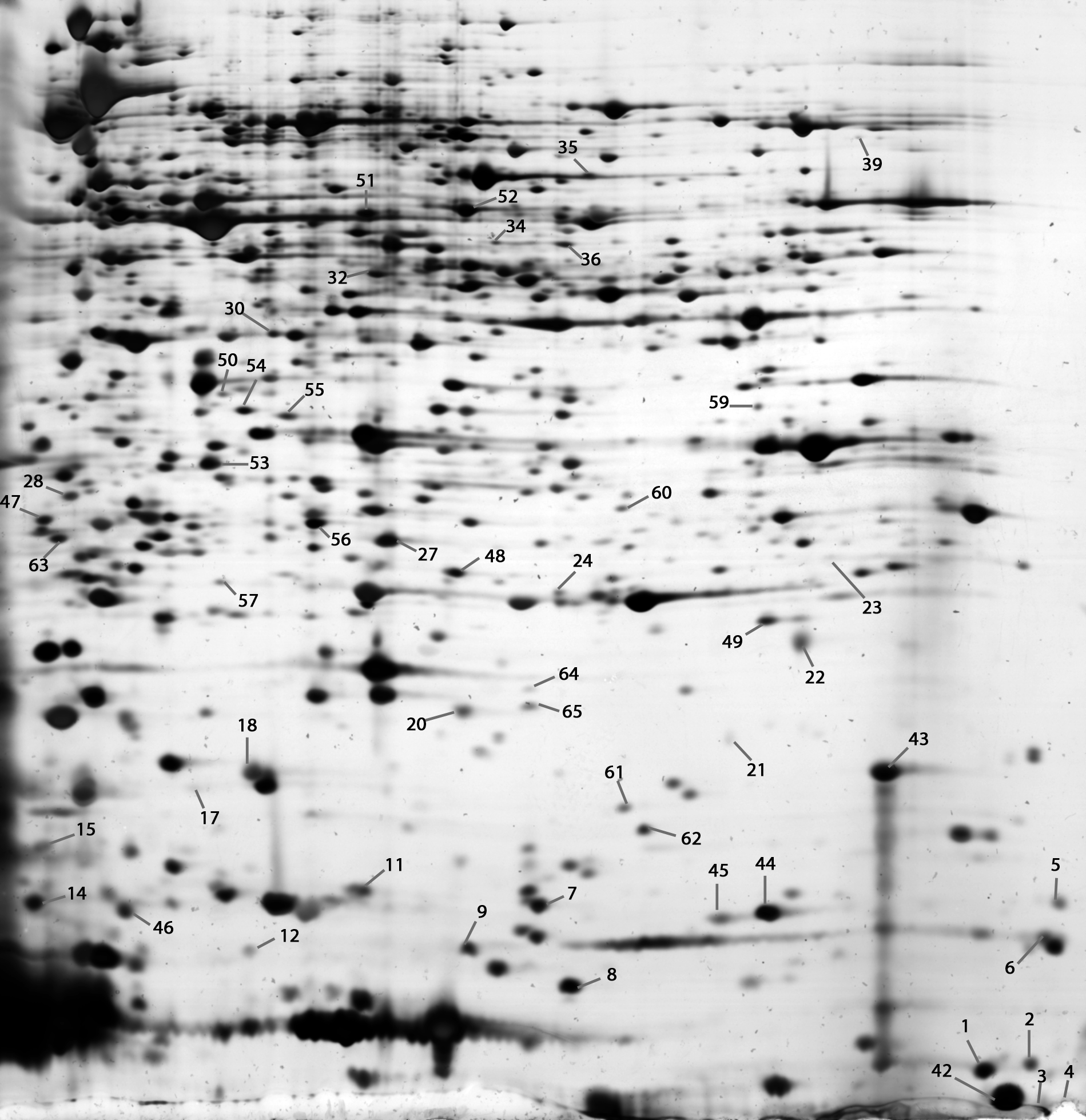

Supplement: Figure S2 — 2-DE map of total cell proteins from P. luminescens LN2 RifR mutant LN2-R2. A representative gel shows the identified differentially expressed protein spots. 350 µg of total cell proteins was loaded onto a 17 cm pH 3–10 NL IPG strip, separated in the second dimension by SDS-polyacrylamide gel electrophoresis on a 12% gel and stained with silver nitrate. (TIF) [file pone.0043114.s002.tif]

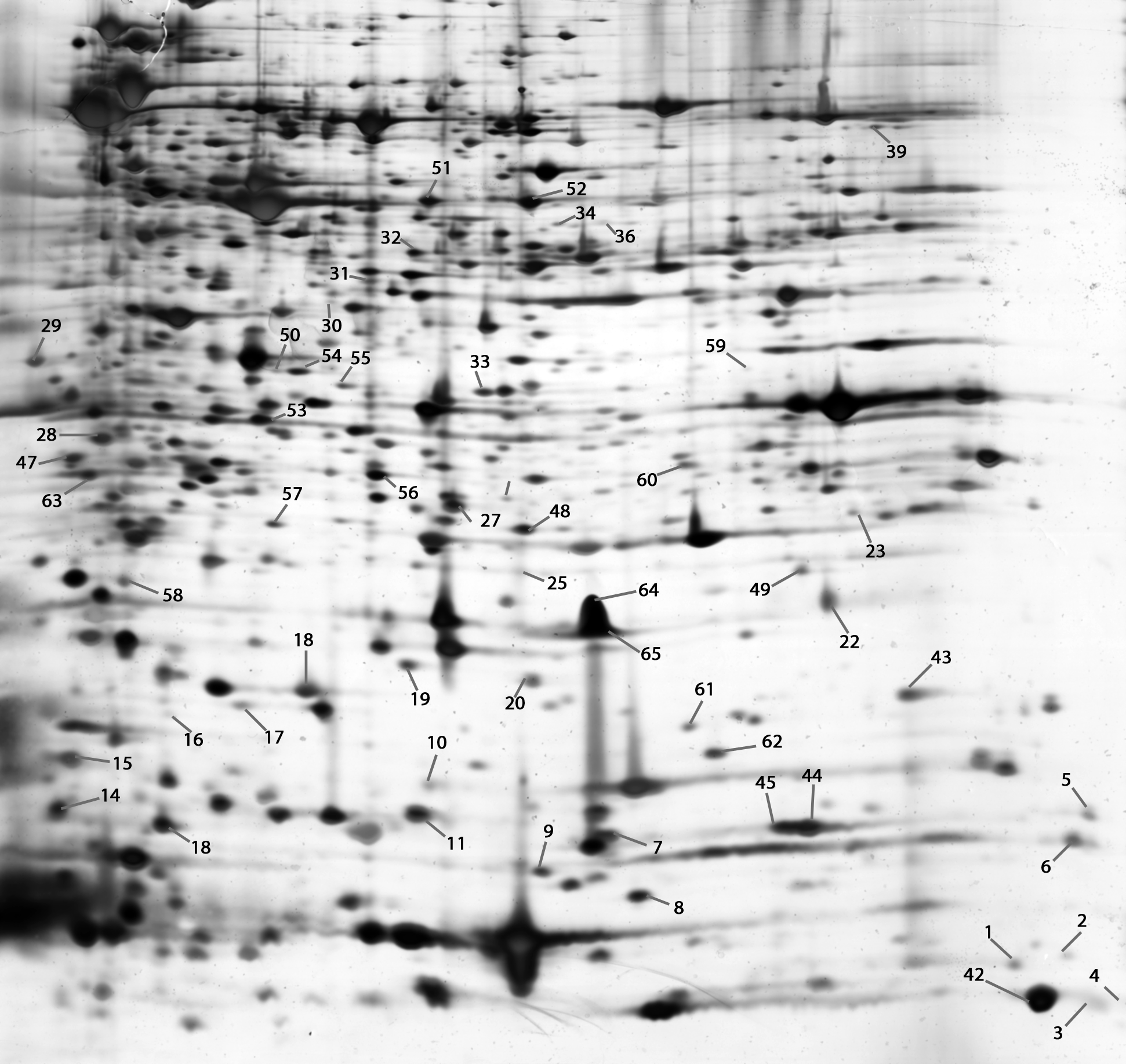

Supplement: Figure S3 — 2-DE map of total cell proteins from P. luminescens LN2 RifR mutant LN2-R16. A representative gel shows the identified differentially expressed protein spots. 350 µg of total cell proteins was loaded onto a 17 cm pH 3–10 NL IPG strip, separated in the second dimension by SDS-polyacrylamide gel electrophoresis on a 12% gel and stained with silver nitrate. (TIF) [file pone.0043114.s003.tif]

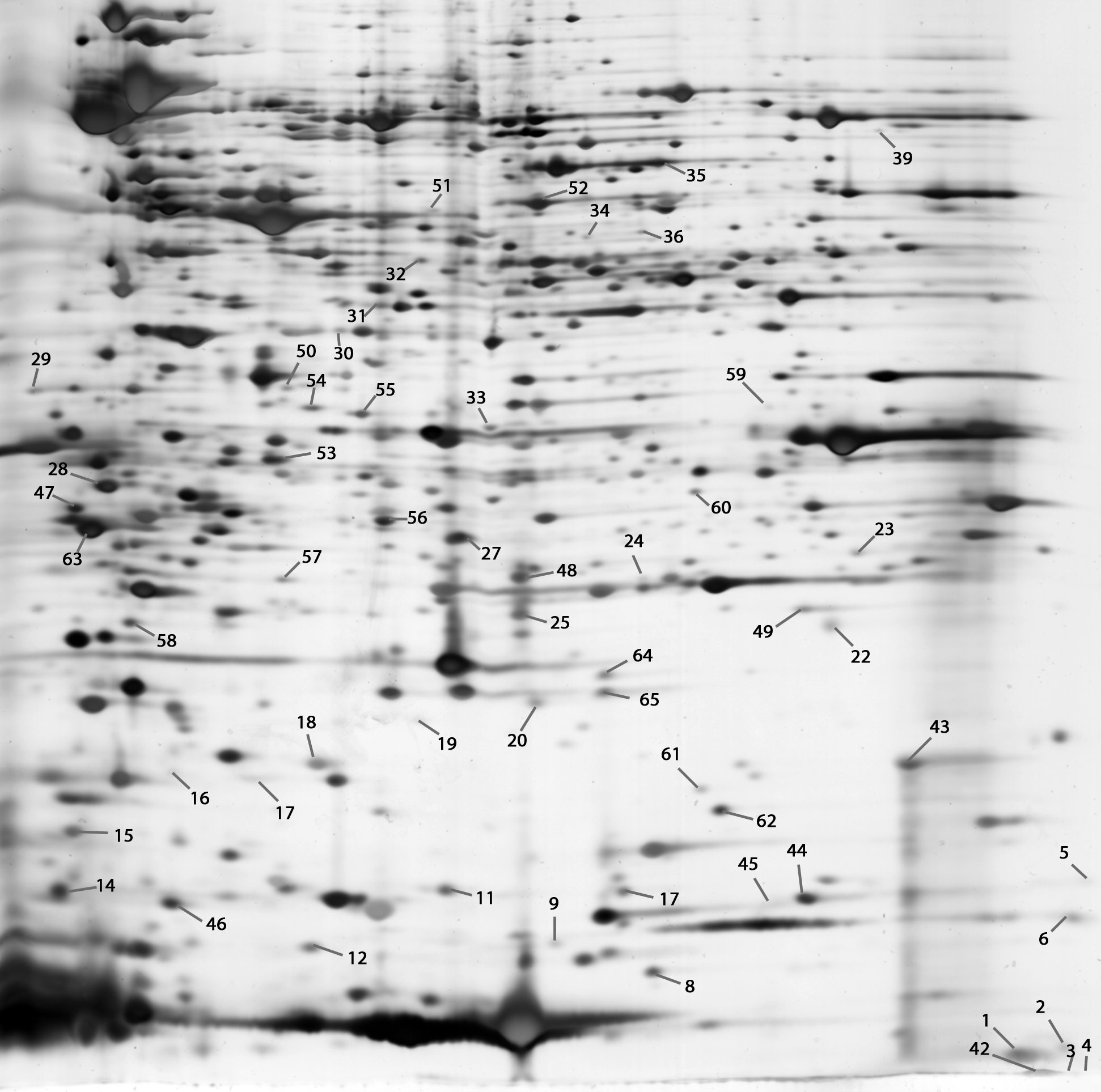

Supplement: Figure S4 — 2-DE map of total cell proteins from P. luminescens LN2 RifR mutant LN2-R31. A representative gel shows the identified differentially expressed protein spots. 350 µg of total cell proteins was loaded onto a 17 cm pH 3–10 NL IPG strip, separated in the second dimension by SDS-polyacrylamide gel electrophoresis on a 12% gel and stained with silver nitrate. (TIF) [file pone.0043114.s004.tif]

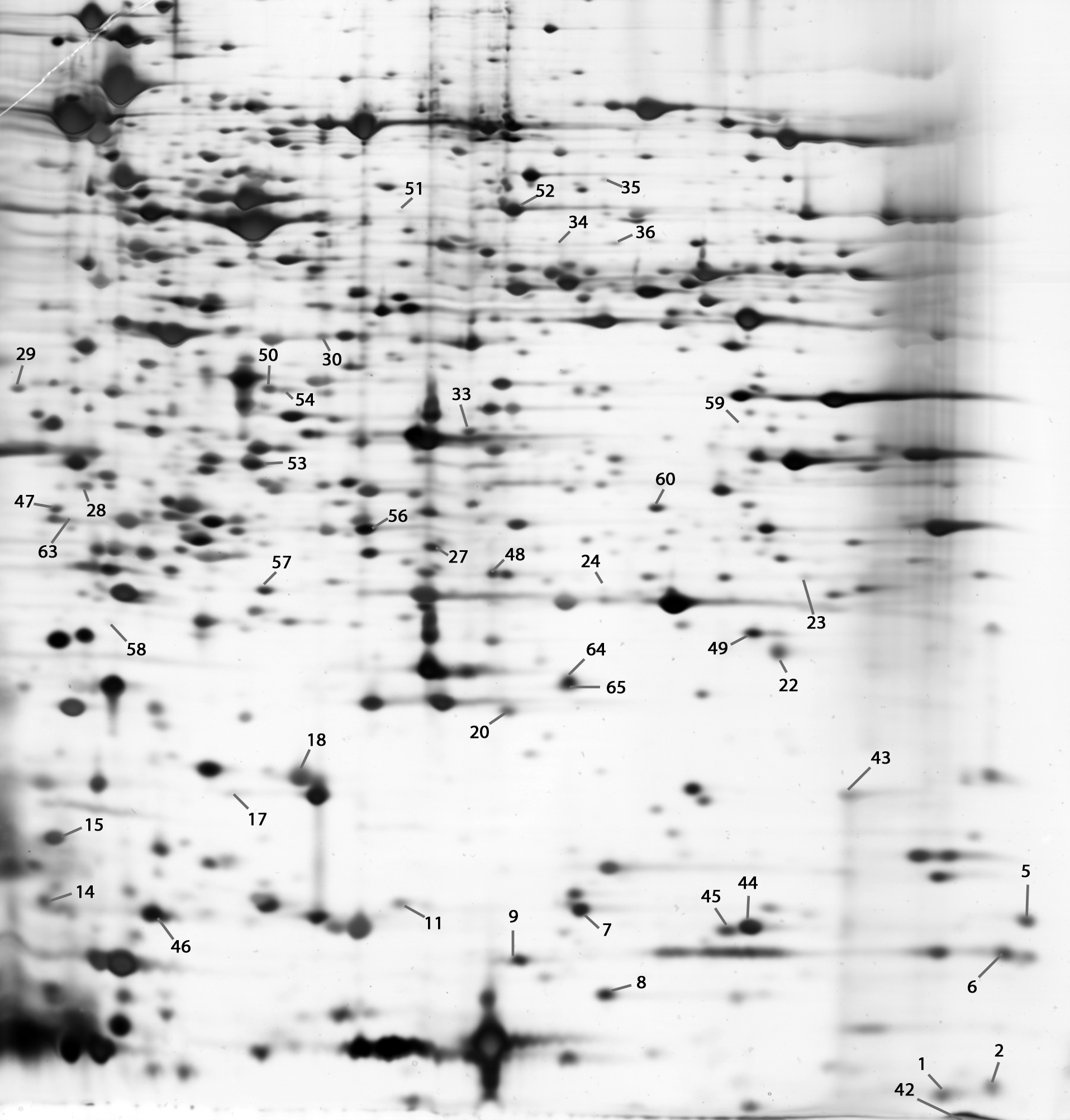

Supplement: Figure S5 — 2-DE map of total cell proteins from P. luminescens LN2 RifR mutant LN2-R33. A representative gel shows the identified differentially expressed protein spots. 350 µg of total cell proteins was loaded onto a 17 cm pH 3–10 NL IPG strip, separated in the second dimension by SDS-polyacrylamide gel electrophoresis on a 12% gel and stained with silver nitrate. (TIF) [file pone.0043114.s005.tif]
