# Supplementary material for: Genetic and Proteomic Characterization of rpoB Mutations and Their Effect on Nematicidal Activity in Photorhabdus luminescens LN2
Source: PLoS One. 2012 Aug 17;7(8):e43114. doi: 10.1371/journal.pone.0043114 (PMC3422287; doi:10.1371/journal.pone.0043114)
Supplement: Table S1 — Oligonucleotide sequences used to generate Photorhabdus luminescens LN2 mutant constructs in this study. (DOC) [file pone.0043114.s006.doc]

**Table S1.** Oligonucleotide sequences used to generate *Photorhabdus luminescens* LN2 mutant constructs in this study.

| *Gene*-Primer | Sequence (5' – 3') |
| --- | --- |
|  |  |
| *camR*-P3 | TTGATCGGCACGTAAGAGGT |
| *camR*-P4 | AATTTCTGCCATTCATCCGC |
|  |  |
| *namB*-P1 | GGATCCAGATAGTTATGTTTGGTGGG |
| *namB*-P2 | ACCTCTTACGTGCCGATCAATATGCCCCACACTGTCTCCA |
| *namB*-P5 | GCGGATGAATGGCAGAAATTCTGAAAGTTGTGTGTAGGGT |
| *namB*-P6 | ACTAGTACAGAAGGCAAGAATGAAGG |
|  |  |
| *dsbA*-P1 | CCCGGGAGGCTGATTTACCTGTTGCG |
| *dsbA*-P2 | ACCTCTTACGTGCCGATCAAGCCATCACCATTCCGACTAA |
| *dsbA*-P5 | GCGGATGAATGGCAGAAATTCGGCTGAGGATTATGATGCT |
| *dsbA*-P6 | CCCGGGAATACGGCGAGAGGGTTGAG |
|  |  |
| *rhlE*-P1 | GGATCC GTATAGGCATCTGTTGGGGC |
| *rhlE*-P2 | ACCTCTTACGTGCCGATCAAGTTTTACCCGTGCCTGTTTG |
| *rhlE*-P5 | GCGGATGAATGGCAGAAATTCAGAAAAACAGCCATCGTCG |
| *rhlE*-P6 | CGCAAATAACCAAGCCACTG |
